# Expansion of epigenetic alterations in *EFEMP1* promoter predicts malignant formation in pancreatobiliary intraductal papillary mucinous neoplasms

**DOI:** 10.1007/s00432-016-2164-x

**Published:** 2016-04-19

**Authors:** Kazuhiro Yoshida, Takeshi Nagasaka, Yuzo Umeda, Takehiro Tanaka, Keisuke Kimura, Fumitaka Taniguchi, Tomokazu Fuji, Kunitoshi Shigeyasu, Yoshiko Mori, Hiroyuki Yanai, Takahito Yagi, Ajay Goel, Toshiyoshi Fujiwara

**Affiliations:** Department of Gastroenterological Surgery, Okayama University Graduate School of Medicine, Dentistry and Pharmaceutical Sciences, 2-5-1 Shikata-cho, Kita-ku, Okayama City, Okayama 700-8558 Japan; Department of Pathology, Okayama University Graduate School of Medicine, Dentistry and Pharmaceutical Sciences, Okayama City, Okayama 700-8558 Japan; Center for Gastrointestinal Cancer Research, Center for Epigenetics, Cancer Prevention and Cancer Genomics, Baylor Research Institute and Charles A Sammons Cancer Center, Baylor University Medical Center, Dallas, TX 75246 USA

**Keywords:** Mucinous neoplasms, Methylation, Epigenetics, EFEMP1, Invasive carcinoma, Dysplasia

## Abstract

**Purpose:**

Although limited understanding exists for the presence of specific genetic mutations and aberrantly methylated genes in pancreatobiliary intraductal papillary mucinous neoplasms (IPMNs), the fundamental understanding of the dynamics of methylation expansion across CpG dinucleotides in specific gene promoters during carcinogenesis remains unexplored. Expansion of DNA methylation in some gene promoter regions, such as *EFEMP1*, one of the fibulin family, with tumor progression has been reported in several malignancies. We hypothesized that DNA hypermethylation in *EFEMP1* promoter would expand with the tumor grade of IPMN.

**Methods:**

A sample of 65 IPMNs and 30 normal pancreatic tissues was analyzed. IPMNs were divided into the following three subsets according to pathological findings: 31 with low-grade dysplasia (low grade), 11 with high-grade dysplasia (high grade), and 23 with associated invasive carcinoma (invasive Ca). Mutations in the *KRAS* or *GNAS* genes were analyzed by Sanger sequencing, and methylation status of two discrete regions within the *EFEMP1* promoter, namely region 1 and region 2, was analyzed by bisulfite sequencing and fluorescent high-sensitive assay for bisulfite DNA (Hi-SA). Expression status of EFEMP1 was investigated by immunohistochemistry (IHC).

**Results:**

*KRAS* mutations were detected in 39, 55, and 70 % of low-grade, high-grade, and invasive Ca, respectively. *GNAS* mutations were observed in 32, 55, and 22 % of low-grade, high-grade, and invasive Ca, respectively. The methylation of individual regions (region 1 or 2) in the *EFEMP1* promoter was observed in 84, 91, and 87 % of low-grade, high-grade, and invasive Ca, respectively. However, simultaneous methylation of both regions (extensive methylation) was exclusively detected in 35 % of invasive Ca (*p* = 0.001) and five of eight IPMNs (63 %) with extensive methylation, whereas 20 of 57 (35.1 %) tumors of unmethylation or partial methylation of the *EFEMP1* promoter region showed weak staining EFEMP1 in extracellular matrix (*p* = 0.422). In addition, extensive *EFEMP1* methylation was particularly present in malignant tumors without *GNAS* mutations and associated with disease-free survival of patients with IPMNs (*p* < 0.0001).

**Conclusions:**

Extensive methylation of the *EFEMP1* gene promoter can discriminate invasive from benign IPMNs with superior accuracy owing to their stepwise accumulation of tumor progression.

**Electronic supplementary material:**

The online version of this article (doi:10.1007/s00432-016-2164-x) contains supplementary material, which is available to authorized users.

## Introduction

Pancreatic intraductal papillary mucinous neoplasms (IPMNs) are precursor lesions characterized by an atypical degree of intraductal proliferation of neoplastic mucinous cells arising in the pancreatic duct (Das et al. 2013; Matthaei et al. 2012; Tanaka et al. 2012; Wasif et al. 2010). Histologically, IPMNs may progress from low-grade to high-grade dysplasia and finally to an invasive carcinoma (Das et al. 2013; Farrell and Brugge 2002; Salvia et al. 2004), canonical to the adenoma-carcinoma sequence in colorectal cancer and pancreatic ductal adenocarcinoma (PDAC) (Wasif et al. 2010). While IPMNs with invasive carcinoma have a poor 5-year survival ratio of 33–43 %, patients with resected IPMNs without any invasive cancer features generally have a better 5-year survival ratio of 77–94 % (Chari et al. 2002; Das et al. 2013; Farrell and Brugge 2002; Maire et al. 2002; Raimondo et al. 2002; Sohn et al. 2004), highlighting the importance of efficient diagnosis of IPMNs with invasive carcinoma. Therefore, preoperative identification of dysplastic IPMNs is challenging even with a multimodality approach including radiographic imaging, endoscopic ultrasound-guided fine-needle aspiration (EUSFNA), cytological examination, and tumor markers (Schoedel et al. 2006). This problem has prompted the development of other analytical tools, including genomic biomarkers that can predict IPMNs at a high risk of developing dysplasia with malignant potential (Schoedel et al. 2006).

Applying molecular techniques to evaluate surgical and cytological specimens is evolving in conjunction with our understanding of the IPMN molecular makeup (Schoedel et al. 2006). Studies aimed at characterizing genetic profiles in IPMNs have identified activating mutations of *KRAS* and *GNAS* oncogenes and inactivating mutations in *RNF43*, *CDKN2A/p16*, and *TP53* tumor suppressor genes (Amato et al. 2014; Cooper et al. 2013; Dal Molin et al. 2013; Furukawa et al. 2011; Kanda et al. 2013; Komatsu et al. 2014; Schonleben et al. 2007, 2008; Sessa et al. 1994). Collectively, these studies underscored the importance of genetic alterations in IPMN progression; however, the prevalence of such genetic events generally occurs at lower frequencies than in PDAC (Adsay 2002; Cooper et al. 2013; Kanda et al. 2013; Sato and Goggins 2006).

Similar to other cancers, epigenetic alterations, such as promoter hypermethylation of tumor suppressor genes, are considered a critical process in IPMN development (Sato and Goggins 2006). Results suggest a gradual expansion of methylation across CpG islands in *MGMT, RASSF2*, and *SFRP2* promoters during colorectal cancer progression and highlighted their potential role as biomarkers for diagnosis and disease prediction for specific cancer types (Nagasaka et al. 2008, 2009; Takeda et al. 2011). In this study, we evaluated the methylation status of the epidermal growth factor-containing fibulin-like extracellular matrix protein 1 gene (*EFEMP1*, alternative annotation is *FIbulin3*), a member of the fibulin family of extracellular matrix (ECM) proteins. EFEMP1 is involved in malignant transformation through modulation of cell proliferation, angiogenesis, and invasion in a tissue-dependent manner (Kobayashi et al. 2007; Sadr-Nabavi et al. 2009; Wang et al. 2010, 2012; Yang et al. 2013; Yue et al. 2007; Zhu et al. 2014), and alterations in this gene expression have often been linked to aberrant DNA methylation (Nomoto et al. 2010; Sadr-Nabavi et al. 2009; Wang et al. 2010, 2012; Yang et al. 2013; Yue et al. 2007; Zhu et al. 2014). Moreover, recent studies have reported an association between a reduction in protein expression and *EFEMP1* methylation expansion in breast and lung cancers using immunohistochemistry and sequencing approaches (Chen et al. 2014; Sadr-Nabavi et al. 2009). For these reasons, the presence or absence of methylation and gradual expansion of specific gene promoter methylation may help diagnose and differentiate invasive carcinoma from normal adjacent tissues and dysplastic lesions.

To systematically test this hypothesis, we first analyzed mutations in the *KRAS* and *GNAS* genes to confirm the genetic background of IPMNs. Next, to determine whether extensive methylation of candidate genes may serve as a predictive alteration for malignant IPMNs, we performed a comprehensive investigation of the methylation status of *EFEMP1* promoter and examined EFEMP1 expression status by IHC.

## Materials and methods

### Samples and tumor classifications

Nine tissues from non-necrotic areas of PDAC were frozen immediately at −80 °C, and DNA was extracted from the tissues using QIAamp DNA mini kits (Qiagen, Valencia, CA, USA). DNAs of 21 tissues from non-necrotic areas of PDAC were macrodissected from formalin-fixed, paraffin-embedded (FFPE) specimens. DNAs of IPMNs were macrodissected from 65 patients who underwent surgical resection and who were pathologically diagnosed with IPMNs or invasive IPMNs. All samples were collected from the Okayama University Hospital, Okayama, Japan, between January 2001 and December 2012. Institutional review board approval was granted by the Ethics Committee of the Okayama University, and written informed consent was obtained from all patients to use their tissues for research. The medical records of the patients were retrospectively explored and matched with clinical and pathological data. We defined IPMN classification based on the International Consensus Guidelines from 2012 as follows: MD-IPMNs were characterized by segmental or diffused dilation of the main pancreatic duct by >5 mm in the absence of other causes of obstruction; BD-IPMNs comprised pancreatic cysts of >5 mm in diameter communicating with the main pancreatic duct; finally, mixed-type lesions were those lesions that simultaneously met the criteria of both MD-IPMNs and BD-IPMNs (Tanaka et al. 2012). In addition, we used a revised terminology to classify IPMNs. Formally, IPMNs are classified according to World Health Organization classification based on pathological findings as follows: IPMNs with low-grade dysplasia, intermediate-grade dysplasia, high-grade dysplasia (carcinoma in situ) and associated invasive carcinoma. Based on the revised classification criteria proposed in 2015, IPMNs with intermediate-grade dysplasia are combined together with the ones with low-grade dysplasia and are now called low-grade IMPNs. Therefore, we classified IPMNs according to this revised nomenclature.

### IPMN patients’ characteristics

Using the criteria mentioned above, our sample of 65 IPMNs was classified as follows: 31 (48 %), 11 (17 %), and 23 (35 %) IPMNs were classified as low- or intermediate-grade dysplasia (low grade), high-grade dysplasia (high grade), and invasive Ca, respectively. To clarify the clinicopathological features of IPMNs, statistical analyses were performed between low-grade and high-grade and invasive Ca (Supplementary Table 1). Within the group of 23 invasive Ca, only one case showed a distant metastasis in the liver at surgical resection (and hence classified as stage IV); the remainder of the invasive Ca cases was categorized as stage I (10 of 23; 44 %) and stage II (12 of 23; 52 %, Supplementary Table 2). Disease-free survival (DFS) and overall survival (OS) of IPMNs were estimated according to their clinicopathological characteristics (Supplementary Fig. 1). The median time of the follow-up period of 65 IPMNs after surgical resection was 48 months (range 8–108 months). The lesions categorized as invasive Ca were also classified by the tumor node metastasis classification system (Adsay et al. 2010; Kim et al. 2008).

### Direct sequencing of *KRAS* and *GNAS* mutations in IPMNS tissues

*KRAS* mutations in codons 12 and 13 were determined by the method previously described (Nagasaka et al. 2008, 2009; Takeda et al. 2011). *GNAS* mutations in codon 201 were analyzed by direct sequencing using *GNAS* primers (Supplementary Fig. 2 and Supplementary Table 3).

### Analysis of DNA methylation

**Fig. 1:**
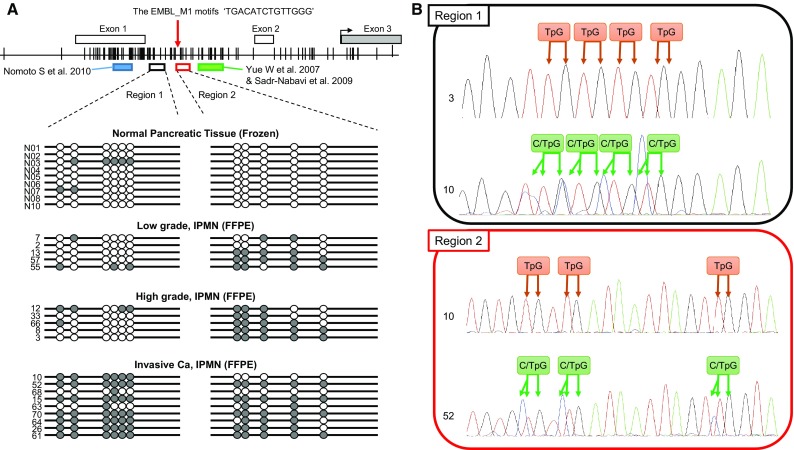
Bisulfite sequencing of the discrete *EFEMP1* gene promoter regions. **a** Schematic representation of the location of discrete *EFEMP1* gene promoter regions and the result of bisulfite sequencing. The* white* and* gray boxes* denote untranslated and translated exon in the *EFEMP1* gene, respectively. The* red allow* indicates the location of the EMBL_M1 motifs ‘TGACATCTGTTGGG’, a candidate of CTCF biding site. The* black arrow* indicates the transcriptional starting site. The* blue box* indicates the regions of which methylation status was analyzed by Nomoto et al. (2010). The* green box* indicates the regions of which methylation status was analyzed by Yue et al. (2007) and Sadr-Nabavi et al. (2009).* Vertical lines* indicate CpG sites; *white circles* represent unmethylated CpGs; and *gray circles* represent methylated and unmethylated CpGs observed by bisulfite direct sequencing. **b** Examples of bisulfite sequencing in region 1 and 2. Each CpG was categorized as unmethylated or methylated CpG. TpG denotes the CpG site consisting of unmethylated CpG only. C/TpG denotes the CpG site consisting of both methylated (CpG) and unmethylated CpGs (TpG)

DNA was subjected to sodium bisulfite modification using the EZ DNA Methylation Kit (ZYMO Research, Irvine, CA). As shown in Fig. 1, methylation status of the *EFEMP1* gene promoter was studied at various regions in previous studies (Kobayashi et al. 2007; Nomoto et al. 2010; Sadr-Nabavi et al. 2009; Wang et al. 2010, 2012; Yang et al. 2013; Yue et al. 2007; Zhu et al. 2014). In this study, we searched CTCF biding sites by CTCFBSDB 2.0 (http://insulatordb.uthsc.edu/) in promoter region of the *EFEMP1* gene and found a CTCF biding site ‘TGACATCTGTTGGG,’ called as the EMBL_M1 motifs (Schmidt et al. 2012). CTCF, also known as CCCTC-binding factor, is a transcription factor involved in many cellular processes, including transcriptional regulation, insulator activity, V(D)J recombination, and regulation of chromatin architecture (Chaumeil and Skok 2012; Phillips and Corces 2009). Interestingly, this biding site was between the two regions which were analyzed by previous studies as mentioned above. For the reason, we divided the *EFEMP1* gene promoter into two regions (regions 1 and 2, Fig. 1), which were located as dividing line on the CTCF biding site, and analyzed by a fluorescence high-sensitive assay (Hi-SA) using bisulfite-modified DNA template as previously described (Nagasaka et al. 2009). The sense and antisense nonspecific primers and internal methylation-specific primers with enhanced sensitivity for polymerase chain reaction (PCR) amplification have been described previously (Nagasaka et al. 2009) and are shown in Supplementary Table 3. PCR products digested with *HhaI* (New England BioLabs, Massachusetts, USA) were loaded simultaneously onto an ABI 310R or 31000 Genetic Analyzer (Applied Biosystems, California, USA). Signals from individual PCR products were distinguished by the unique fluorescent PCR signal from each target and their fragment length, and the data were analyzed using GeneMapper software version 4.0 (Applied Biosystems, California, USA). In this study, the percentages of methylated *HhaI* sites were calculated by determining the ratios between the *HhaI*-cleaved PCR product and the total amount of PCR product in each loci, and methylation positivity was defined as a proportion of >1.0 % of methylated *HhaI* sites. Direct sequencing of the two regions in the *EFEMP1* promoter was performed by PCR products obtained from bisulfite DNA extracted from normal pancreatic tissues and IPMNs. Primer sequences are shown in Supplementary Table 3.

### Immunohistochemical analysis

EFEMP1 localization was performed by immunohistochemical (IHC). A sample of 65 IPMNs was available for IHC staining for EFEMP1 protein expression analysis. Staining was carried out manually with FFPE tissues. Thin (5 µm) sections of representative blocks were deparaffinized and dehydrated using gradient solvents. Following antigen retrieval in the citrate buffer (pH 6.0), endogenous peroxidase was blocked with 3 % H_2_O_2_. Thereafter, slides were incubated overnight in the presence of a purified mouse anti-human EFEMP1 monoclonal antibody (sc-33722, Santa Cruz, Dallas, TX, USA; dilution 1:250). Further incubation was carried out with a secondary antibody and the avidin–biotin–peroxidase complex (Vector Laboratories, Burlingame, CA, USA) and then incubated with biotinyl-tyramide followed by streptavidin-peroxidase. Diaminobenzidine was used as a chromogen and hematoxylin as a nuclear counterstain.

EFEMP1 was detectable in normal pancreatic tissue; weak staining was detected in islets of Langerhans, whereas intense staining was observed in the peripheral nerve fiber. IHC results were interpreted by pathologists blinded to the corresponding clinicopathological data. The expression status of EFEMP1 was evaluated by an immunoreactive score (IRS), which was calculated by scoring of the percentage of positive cells and their expression intensities. The percentage of positive ECM staining was rated as described previously and as follows: 1 = 0–10 %, 2 = 11–50 %, 3 = 51–80 %, and 4 = 81–100 % (Sadr-Nabavi et al. 2009). Staining intensity was scored as follows: 1 = weak, 2 = moderate, and 3 = intensive. All IPMNs were categorized into four subsets by IRS score as follows: 0–1 = no staining, 2–3 = weak staining, 4–8 = moderate staining, and 9–12 = strong staining (Remmele and Stegner 1987).

### Statistical analyses

All statistical analyses were performed using EZR (Saitama Medical Center, Jichi Medical University), which is a graphical user interface for R (The R Foundation for Statistical Computing, version 2.13.0). First, methylation levels were analyzed as continuous variables. Next, the methylation status was analyzed as a categorical variable (positive, methylation level >1.0 %; negative, methylation level ≤1.0 %), as described previously. Each IPMN specimen was given a numerical score so as to reflect the number of methylated loci. Categorical variables were compared by Fisher’s exact test. Differences between continuous variables were determined using the Mann–Whitney *U* test or the Kruskal–Wallis test. Multiple comparisons were performed using the Steel–Dwass test. OS was calculated from the date of surgical resection to the date of death due to IPMNS or last follow-up for censored patients. DFS was calculated from the date of surgical resection to the date of the first documentation of local, regional, or distant relapse, appearance of a second primary lesion by computed tomography and/or magnetic resonance imaging routinely performed per 6 months. OS and DFS were univariately estimate with the Kaplan–Meier method. All *p* values reported were calculated by two-sided tests, and values <0.05 were considered statistically significant.

## Results

### KRAS *and* GNAS *mutations in IPMNs*

To evaluate the genetic background in our current cohort, we analyzed mutations in *KRAS* and *GNAS* genes as shown in Supplementary Figure 2. *KRAS* mutations were detected in 33 IPMNs (51 %), and the spectrum of relative frequencies of individual mutations was 5 (15 % of *KRAS* mutants), 12 (36 %), and 16 (49 %) for G12R, G12D, and G12V mutations, respectively (Supplementary Tables 1 and 2). Meanwhile, 19 IPMNs (29 %) harbored *GNAS* mutations, and the following mutations were primarily found in codon 201: R201H, 5 IPMNs (26 %); R201C, 13 IPMNs (68 %); and R201S, 1 IPMNs (5 %) (Table 2). One R201S was a novel mutation, which had not been previously described for IPMNs. *KRAS* mutation frequency tended to increase with IPMN grade: 12 of 31 were low grade (39 %), 6 of 11 were high grade (55 %), and 16 of 23 were invasive Ca (70 %, *p* = 0.084; low-grade vs. high-grade/invasive Ca). In contrast, *GNAS* mutations were constantly observed, with 10 of 32 (32 %) in low-grade and 12 of 33 (36 %) in high-grade and invasive Ca. Concurrent *KRAS* and *GNAS* mutations were observed in 11 IPMNs (17 %), *KRAS* mutation were present only in 22 IPMNs (34 %), *GNAS* mutations only in 8 IPMNs (12 %), and wild-type status of both genes was observed in 24 IPMNs (37 %, Supplementary Fig. 2C).

We assessed DFS and OS according to *KRAS* or *GNAS* mutation status. Although *KRAS* mutations were more frequently observed in invasive Ca, there was no difference in outcome between *KRAS* mutants and wild type (Supplementary Fig. 2E and F). In contrast, *GNAS* mutations were less frequently observed in invasive Ca compared with low and high grade, and there was no difference in outcome between *GNAS* mutants and wild type (Supplementary Fig. 2G and H).

### *Methylation profiles of* EFEMP1 *promoter in IPMNs*

**Fig. 2:**
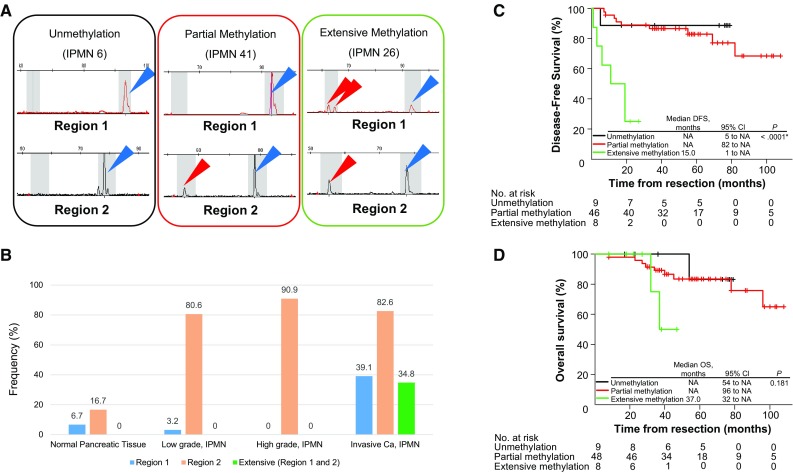
Methylation analysis of *EFEMP1*. **a** Results of *EFEMP1* methylation by fluorescent Hi-SA. *Blue arrows* represent PCR fragments with non-methylation in HhaI sites. *Red arrows* represent methylated PCR fragments cleaved by HhaI. **b** Frequencies of *EFEMP1* methylation according to pathological findings. Kaplan–Meier survival curves for disease-free survival (**c**), excluding a patient with remaining cancer at the resected margin, and overall survival (**d**) according to *EFEMP1* promoter methylation status

We investigated the methylation status of discrete regions in the *EFEMP1* promoter in 65 IPMNs and 30 normal pancreatic tissues obtained from PDAC patients. The location of the *EFEMP1* gene and a panel of representative bisulfite sequencing and fluorescent Hi-SA results are depicted in Figs. 1 and 2a, respectively. Methylation status in the discrete regions obtained from fluorescent Hi-SA was analyzed as the categorical variable. Figure 2b presents methylation frequency in each discrete region according to pathological features, and Table 1 presents the correlation between the methylation status of *EFEMP1* and the clinical and pathological features of IPMNs. The region-1 methylation was frequently observed in invasive Ca (*p* = 0.0016), whereas the region-2 methylation was commonly observed in IPMN (>80 % of IPMNs) but less frequently in normal pancreatic tissues (<20 %). Another interesting feature of IPMNs with extensive *EFEMP1* methylation was histologic subtype, in which extensive *EFEMP*1 methylation was frequently observed in pancreatobiliary type lesions (4 of 6; 67 %, *p* = 0.027).

**Table 1:** Correlation between characteristics of patients and *EFEMP1* methylation status

	All no. (%)	Methylation status of *EFEMP1*—no (%)									
		Unmethylation *n* = 9 (%)	Partial methylation *n* = 48 (%)	Extensive methylation *n* = 8 (%)	*p* value	Region 1 unmethylation *n* = 55 (%)	Region 1 methylation *n* = 10 (%)	*p* value	Region 2 unmethylation *n* = 11 (%)	Region 2 methylation *n* = 54 (%)	*p* value
*Age (years)*											
Median (range)	68 (39–88)	66 (54–82)	70 (39–81)	71 (51–88)	0.482^a^	63 (39–82)	70 (51–88)	0.716^c^	68 (54–82)	70 (39–88)	0.234^c^
*Gender*											
Female	21 (32)	2 (22)	17 (35)	2 (25)	0.753^b^	19 (35)	2 (20)	0.479^d^	2 (18)	19 (35)	0.48^d^
Male	44 (68)	7 (78)	31 (65)	6 (75)		36 (76)	8 (80)		9 (82)	35 (65)	
*Location*											
Head, neck and uncinate	34 (52)	6 (67)	24 (50)	4 (50)	0.344^b^	29 (53)	5 (50)	1^d^	7 (64)	27 (50)	0.182^d^
Body and tail	29 (45)	2 (22)	23 (48)	4 (50)		24 (44)	5 (50)		3 (27)	26 (48)	
Whole pancreas	2 (3)	1 (11)	1 (2)	0 (0)		2 (4)	0 (0)		1 (9)	1 (2)	
Duct involvement*											
MD-IPMN	21 (33)	4 (44)	14 (30)	3 (38)	0.896^b^	16 (30)	5 (50)	0.473^d^	6 (55)	15 (28)	0.261^d^
BD-IPMN	20 (31)	3 (33)	15 (32)	2 (25)		18 (33)	2 (20)		3 (27)	17 (32)	
Mixed type	23 (36)	2 (22)	18 (38)	3 (38		20 (37)	3 (30)		2 (18)	21 (40)	
Cystic size** (cm)											
Median size (range)	2.7 (0.5–7.5)	2.6 (0.5–6.7)	2.7 (0.5–7.5)	3.9 (0.5–6.1)	0.872^a^	2.7 (0.5–7.5)	2.7 (0.5–6.1)	0.958^c^	1.7 (0.5–6.7)	2.9 (0.5–7.5)	0.517^c^
<3.0 cm	35 (54)	5 (56)	27 (56)	3 (38)	0.657^b^	30 (55)	5 (50)	1^d^	7 (64)	28 (52)	0.526^d^
>3.0 cm	30 (46)	4 (44)	21 (44)	5 (63)		25 (46)	5 (50)		4 (36)	26 (48)	
*Main duct diameter (cm)*											
Median size (range)	0.7 (0.2–7.1)	0.6 (0.2–6.7)	0.7 (0.2–7.1)	0.6 (0.2–4.0)	0.822^a^	2.7 (0.5–7.5)	2.7 (0.5–6.2)	0.82^d^	0.7 (0.2–6.7)	0.7 (0.2–7.1)	0.441^d^
<1.0 cm	45 (69)	6 (67)	33 (69)	6 (75)	1^b^	39 (71)	6 (60)	0.482^b^	6 (54)	39 (72)	0.292^b^
>1.0 cm	20 (31)	3 (33)	15 (31)	2 (25)		16 (29)	4 (40)		5 (46)	15 (28)	
*Presence of mural nodule*											
Yes	38 (59)	5 (56)	25 (52)	8 (100)	**0.032** ^**a**^	29 (53)	9 (90)	**0.037** ^**a**^	6 (55)	32 (59)	1^a^
No	27 (42)	4 (44)	23 (48)	0 (0)		26 (47)	1 (10)		5 (46)	22 (41)	
*Histologic subtype*											
Gastric	33 (51)	6 (67)	25 (52)	2 (25)	**0.027** ^**a**^	31 (56)	2 (20)	**0.006** ^**a**^	6 (55)	27 (50)	0.756^a^
Intestinal	25 (39)	3 (33)	20 (42)	2 (25)		21 (38)	4 (40)		5 (46)	20 (37)	
Pancreatobillary	6 (9)	0 (0)	2 (4)	4 (50)		2 (4)	4 (40)		0 (0)	6 (11)	
Oncocytic	1 (2)	0 (0)	1 (2)	0 (0)		1 (2)	0 (0)		0 (0)	1 (2)	
*Atypical grade*											
Low grade	31 (48)	5 (56)	26 (54)	0 (0)	**0.0016** ^**b**^	30 (55)	1 (10)	**0.00044** ^**d**^	6 (55)	25 (46)	0.908^d^
High grade	11 (17)	1 (11)	11 (21)	0 (0)		11 (20)	0 (0)		1 (9)	10 (19)	
Invasive Ca	23 (35)	3 (33)	12 (25)	8 (100)		14 (26)	9 (90)		4 (36)	19 (35)	
*KRAS*											
Mutant	33 (51)	3 (33)	25 (52)	5 (63)	0.471^b^	26 (47)	7 (70)	0.303^d^	5 (46)	28 (52)	0.751^d^
Wild type	32 (49)	6 (67)	23 (48)	3 (38)		29 (53)	3 (30)		6 (55)	26 (48)	
*GNAS*											
Mutant	19 (29)	4 (44)	15 (31)	0 (0)	0.102^b^	19 (35)	0 (0)	**0.028** ^**d**^	4 (36)	15 (28)	0.718^d^
Wild type	46 (71)	5 (56)	33 (69)	8 (100)		36 (66)	10 (100)		7 (64)	39 (72)	
*Combined mutational type*											
Mutant in the both genes	11 (17)	2 (22)	9 (19)	0 (0)	0.364^b^	11 (20)	0 (0)	0.058^d^	2 (18)	9 (17)	0.899^d^
*KRAS* mutation alone	22 (34)	1 (11)	16 (33)	5 (63)		15 (27)	7 (70)		3 (27)	19 (35)	
*GNAS* mutation alone	8 (12)	2 (22)	6 (13)	0 (0)		8 (15)	0 (0)		2 (18)	6 (11)	
Wild type in the both genes	24 (37)	4 (44)	17 (35)	3 (38)		21 (38)	3 (30)		4 (36)	20 (37)	

*Low grade* Low-grade dysplasia, *high grade* high-grade dysplasia, *invasive Ca* invasive carcinoma, *MD-IPMN* main duct type IPMN, *BD-IPMN* branch duct type IPMN

* One unknown case was excluded

** Cystic size reprsented the largest one measured by CT or MRI

^a^ *p* value were calculated among unmethylation, partial methylation and extensive methylation by Kruskal–Wallis test

^b^ *p* value were calculated among unmethylation, partial methylation and extensive methylation by Fisher exact test

^c^ *p* value were calculated between unmethylation and methylation of region 1 or 2 within *EFEMP1* promoter by Mann–Whitney U test

^d^ *p* value were calculated between unmethylation and methylation of region 1 or 2 within *EFEMP1* promoter by Fisher exact test

With respect to *KRAS/GNAS* mutation status, interestingly, IPMNs with methylation of the region-1 never harbored *GNAS* mutations (0 of 10; 0 %, *p* = 0.028), while 7 of 10 IPMNs with methylation of the region-1 harbored *KRAS* mutations (70 %). While methylation of the region-2 promoter was frequently observed (54 of 65 IPMNs [83 %]), no associations were observed among the frequencies of region-2 methylation and any of the clinicopathological factors.

Next, we evaluated correlations between the methylation status of *EFEMP1* promoter and the clinicopathological features of invasive Ca (Supplementary Table 3). However, methylation status had no association with any of the features explored in invasive Ca.

Finally, we also assessed DFS and OS according to methylation status in the discrete *EFEMP1* promoter regions. As extensive *EFEMP1* methylation was a specific feature of invasive Ca, IMPNs with extensive *EFEMP1* methylation showed a poor prognosis compared with IMPNs without extensive *EFEMP1* methylation (Fig. 2c, d).

### *Association of* EFEMP1 *promoter methylation and protein expression*

**Fig. 3:**
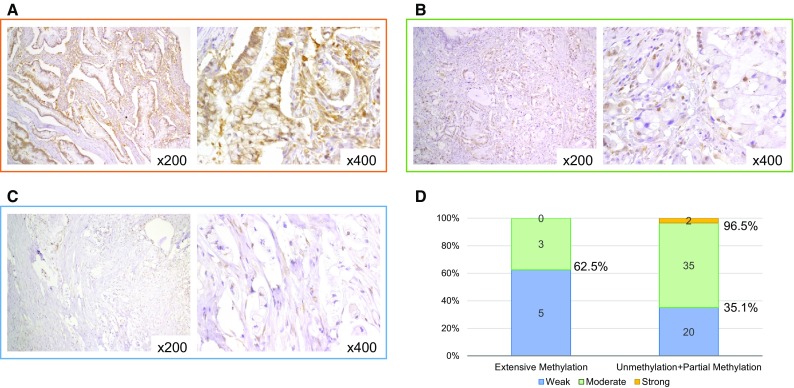
Expression analysis of EFEMP1. IHC staining of EFEMP1 in intraductal papillary mucinous neoplasms with strong staining (**a**), moderate staining (**b**), and weak staining (**c**). Association between *EFEMP1* methylation status and IHC staining (**d**). EFEMP1, epidermal growth factor-containing fibulin-like extracellular matrix protein 1; *IHC* immunohistochemical; *NA* not available

We investigated EFEMP1 protein expression in 65 IPMN tissues. Representative examples of IHC staining results are shown in Fig. 3a–c. Using these criteria, no IPMNs were categorized as having no staining, whereas 25, 38, and 2 IPMNs were deemed to have weak, moderate, and strong staining, respectively. Although five of eight IPMNs (63 %) showed weak staining and extensive methylation of the *EFEMP1* promoter region, no significant differences in the frequencies of EFEMP1 protein expression were observed in IPMNs exhibiting partial methylation or non-methylation in the *EFEMP1* promoter (Fig. 3d).

## Discussion

We have shown for the first time the biological significance of methylation in discrete promoter regions of *EFEMP1* gene in tissue specimens obtained from patients with pancreatobiliary IPMNs, playing an important functional role in malignant transformation by modulating cell proliferation, angiogenesis, and invasion in a tissue-dependent context (Kobayashi et al. 2007); it is a common target of promoter hypermethylation in various tumors (Nomoto et al. 2010; Sadr-Nabavi et al. 2009; Wang et al. 2010, 2012; Yang et al. 2013; Yue et al. 2007; Zhu et al. 2014). Aberrant hypermethylation of the *EFEMP1* promoter region is a potential biomarker. Sadr et al. reported an association between reduction in protein expression and *EFEMP1* methylation expansion in breast cancer using sequencing approaches and IHC (Sadr-Nabavi et al. 2009). In this study, we also examined the association between EFEMP1 expression in ECM by IHC and *EFEMP1* methylation profiles. Unfortunately, although 63 % of IPMNs with extensive methylation of the *EFEMP1* promoter region showed weak staining in ECM, no significant differences in the frequencies of EFEMP1 protein expression were observed in IPMNs exhibiting partial methylation or non-methylation in the *EFEMP1* promoter.

Considering the importance of *EFEMP1*, we hypothesized that gradual expansion of methylation across its promoter during the development of IPMN serves as a biomarker for distinguishing malignant IPMNs from the non-malignant ones. To systematically test this hypothesis, we first analyzed mutations in the *KRAS* and *GNAS* oncogenes to confirm the genetic background of IPMNs. Usually, the methylation pattern in a gene promoter is considered either entirely methylated or non-methylated. However, as demonstrated by bisulfite sequencing, individual CpG residues within the *EFEMP1* promoter were not equally methylated. In this study, region-2 of the *EFEMP1* promoter was more frequently methylated than region-1, and the aberrant methylation tended to spread from region-2 toward region-1 with IPMN progression. More importantly, none of the normal pancreatic tissues, low- or high-grade IMPNs showed extensive methylation in the *EFEMP1* promoter. These characteristics of the *EFEMP1* methylation pattern in IPMN carcinogenesis appeared similar to those of *MGMT*, *SFRP2*, and *RASSF2* in the adenoma-carcinoma sequence of colorectal cancer (Nagasaka et al. 2008, 2009; Takeda et al. 2011). Therefore, the presence or absence of methylation and the gradual expansion of methylation of specific gene promoters may help diagnose and differentiate invasive carcinoma from normal adjacent tissues and dysplastic lesions. This fundamental concept of stepwise expansion of DNA methylation across specific gene promoters during the neoplastic progression of IPMNs remains unexplored and is still an active area of investigation.

*KRAS* and *GNAS* mutations, the most common genetic mutations observed in IPMNs, occurred in the early stages of disease progression. *KRAS* and *GNAS* mutations were also found at codon 12 (a G12D, G12V, or G12R) and codon 201 (an R201H or R201C), respectively. These genetic features are in line with those previously reported in other studies (Amato et al. 2014; Furukawa et al. 2011; Sadr-Nabavi et al. 2009; Wu et al. 2011a, b). This agreement indicates that *KRAS* mutations at codon 12 or *GNAS* mutations at codon 201 could play a key role as the driver of carcinogenesis, providing a selective advantage in tumor formation associated with these IPMNs (Parmigiani et al. 2009). However, the frequencies of both genetic mutations, especially *GNAS* mutations, were different among genetic mutational analyses. In the current study, *GNAS* mutations were observed with similar frequencies in low-grade and invasive Ca. However, Furukawa et al. (2011) reported that *GNAS* mutations were more common in low-grade lesions. In contrast, Amato et al. and other authors showed that *GNAS* mutation frequency tended to increase with tumor progression (Amato et al. 2014; Wu et al. 2011a, b). These differences in observations might partly be due to smaller sample size or variations in the detection technologies used in the studies.

To our knowledge, no studies have been published so far demonstrating a correlation between genetic and epigenetic alterations in IPMNs although several previous reports have revealed that there are distinct patterns of genetic mutations in IPMN subtypes (Amato et al. 2014; Chadwick et al. 2009; Cooper et al. 2013; Dal Molin et al. 2013; Fritz et al. 2009; Komatsu et al. 2014; Mino-Kenudson et al. 2011; Mohri et al. 2012). In this study, extensive methylation of *EFEMP1* was likely to occur in IPMNs without *GNAS* mutations (Table 2). This result might reflect the biological behavior of the *GNAS* gene pathway, which is less aggressive than that of the *KRAS* gene, and further investigation is needed to evaluate the roles of *KRAS* and *GNAS* mutations in IPMN carcinogenesis.

**Table 2:** Methylation status of EFEMP1and Genetic profiles of *KRAS* and *GNAS* in this cohort

Sample no.	Gender	Age	Duct involvement	*KRAS*	*GNAS*	*EFEMP*	
						Region 1	Region 2
*Low grade*							
14	M	71	MD	WT	WT	U	U
44	M	54	Mixed	WT	WT	U	U
47	M	58	MD	WT	WT	U	U
46	M	63	BD	WT	R201C	U	U
7	F	66	BD	WT	WT	U	U
22	F	63	Mixed	WT	WT	U	M
50	M	68	Mixed	WT	WT	U	M
54	F	76	BD	WT	WT	U	M
60	M	66	Mixed	WT	WT	U	M
65	M	63	MD	WT	WT	U	M
39	M	64	BD	WT	R201C	U	M
42	M	59	MD	G12V	WT	M	U
56	M	65	BD	G12V	R201C	U	M
17	F	71	BD	WT	WT	U	M
21	M	67	BD	WT	WT	U	M
31	M	78	MD	WT	WT	U	M
35	F	57	BD	WT	WT	U	M
36	F	62	BD	WT	WT	U	M
2	M	61	Mixed	WT	WT	U	M
58	M	60	BD	WT	R201H	U	M
13	F	74	MD	G12R	WT	U	M
30	M	73	Mixed	G12D	WT	U	M
57	M	74	Mixed	G12R	WT	U	M
67	M	81	BD	G12D	WT	U	M
55	F	71	MD	G12V	R201C	U	M
41	M	50	BD	G12D	WT	U	M
1	F	65	Mixed	G12V	R201H	U	M
69	M	71	Mixed	G12D	R201S	U	M
18	M	68	BD	WT	R201C	U	M
25	M	69	Mixed	G12D	R201C	U	M
9	M	73	Mixed	G12V	R201C	U	M
*High grade*							
12	M	81	MD	WT	WT	U	M
16	M	72	MD	WT	WT	U	M
33	F	70	Mixed	G12D	WT	U	M
43	M	75	Mixed	G12V	R201H	U	M
66	M	79	Mixed	WT	R201C	U	M
19	F	67	Mixed	G12R	WT	U	M
38	M	66	Mixed	G12D	R201H	U	U
8	M	73	Mixed	WT	WT	U	M
3	M	70	MD	G12V	WT	U	M
34	F	59	BD	WT	WT	U	M
53	M	72	MD	WT	R201C	U	M
*Invasive Ca*							
48	F	66	Mixed	WT	WT	U	M
49	M	67	MD	WT	R201C	U	U
10	M	73	MD	G12V	WT	M	U
37	F	59	Unknown	G12V	WT	U	M
4	F	73	MD	G12V	WT	U	M
59	M	66	Mixed	G12V	WT	U	U
6	F	82	MD	G12D	R201C	U	U
20	M	74	MD	G12D	R201H	U	M
32	M	56	BD	WT	WT	U	M
11	M	74	MD	WT	R201C	U	M
52	M	51	Mixed	G12R	WT	M	M
68	M	78	MD	WT	WT	M	M
15	M	67	MD	G12D	WT	M	M
24	F	72	BD	G12V	WT	U	M
51	M	57	MD	G12D	WT	U	M
62	F	81	BD	G12V	R201C	U	M
63	M	75	BD	WT	WT	M	M
70	M	57	Mixed	WT	WT	M	M
5	F	74	MD	G12V	WT	U	M
40	M	39	Mixed	G12V	WT	M	M
64	M	63	BD	G12D	WT	M	M
26	F	79	MD	G12V	WT	M	M
61	M	88	Mixed	G12R	WT	M	M

*M* methylation, *U* unmethylation, MD, MD-IPMN; BD, BD-IPMN; Mixed, Mixed-IPMN

Our study has several limitations. One is the sample size. Another is that, although we examined the correlation between methylation status of our analyzed regions in the *EFEMP* promoter and its expression status by IHC, there was no strong correlation between them; hence, further investigation is needed. Beyond the limitations, we demonstrated that the extensive methylation in the *EFEMP1* promoter could be a useful predictive marker for invasive IPMNs and could serve as a possible means to noninvasively screen for invasive IPMNs using DNA obtained from EUSFNA, pancreatic juice, and fecal samples.

## Electronic supplementary material

Below is the link to the electronic supplementary material.

Supplementary Figure 1 Kaplan–Meier survival curves for DFS and OS according to clinicopathological characteristics. Kaplan–Meier survival curves for DFS (**A, C,** and **E**) excluding a patient with remaining cancer at the resected margin and OS (**B, D,** and **F**) according to atypical grade, invasive carcinoma by stage I and II, and invasive carcinoma by histological type, respectively. DFS, disease-free survival; NA, not available; OS, overall survival (PPTX 91 kb)

Supplementary Figure 2 ***KRAS*** **and** ***GNAS*** **mutations in IPMNs.** (**A**) and (**B**) demonstrate the mutations occurring in *KRAS* codon 12 and *GNAS* codon 201, respectively. (**C**) Venn diagram of IPMNs with *KRAS* mutations, *GNAS* mutations, and concurrent *KRAS* and *GNAS* mutations. (**D**) Population of IPMNs with mutations in both genes, *KRAS* mutation alone, *GNAS* mutation alone, and wild type of both genes. Kaplan–Meier survival curves for disease-free survival (**E** and **G**), excluding a patient with remaining cancer at the resected margin, and overall survival (**F** and **H**) according to *KRAS* and *GNAS* mutation status, respectively. IPMN, intraductal papillary mucinous neoplasm; NA, not available (PPTX 126 kb)

Supplementary material 3 (XLSX 12 kb)

Supplementary material 4 (XLSX 9 kb)

Supplementary material 5 (XLSX 9 kb)
