# Supplementary material for: Expansion of epigenetic alterations in EFEMP1 promoter predicts malignant formation in pancreatobiliary intraductal papillary mucinous neoplasms
Source: J Cancer Res Clin Oncol. 2016 Apr 19;142(7):1557–69. doi: 10.1007/s00432-016-2164-x (PMC4899496; doi:10.1007/s00432-016-2164-x)
Supplement: Supplementary file 1 — Kaplan–Meier survival curves for DFS and OS according to clinicopathological characteristics. Kaplan–Meier survival curves for DFS (A, C, and E) excluding a patient with remaining cancer at the resected margin and OS (B, D, and F) according to atypical grade, invasive carcinoma by stage I and II, and invasive carcinoma by histological type, respectively. DFS, disease-free survival; NA, not available; OS, overall survival (PPTX 91 kb) [file 432_2016_2164_MOESM1_ESM.pptx]

## Slide 1
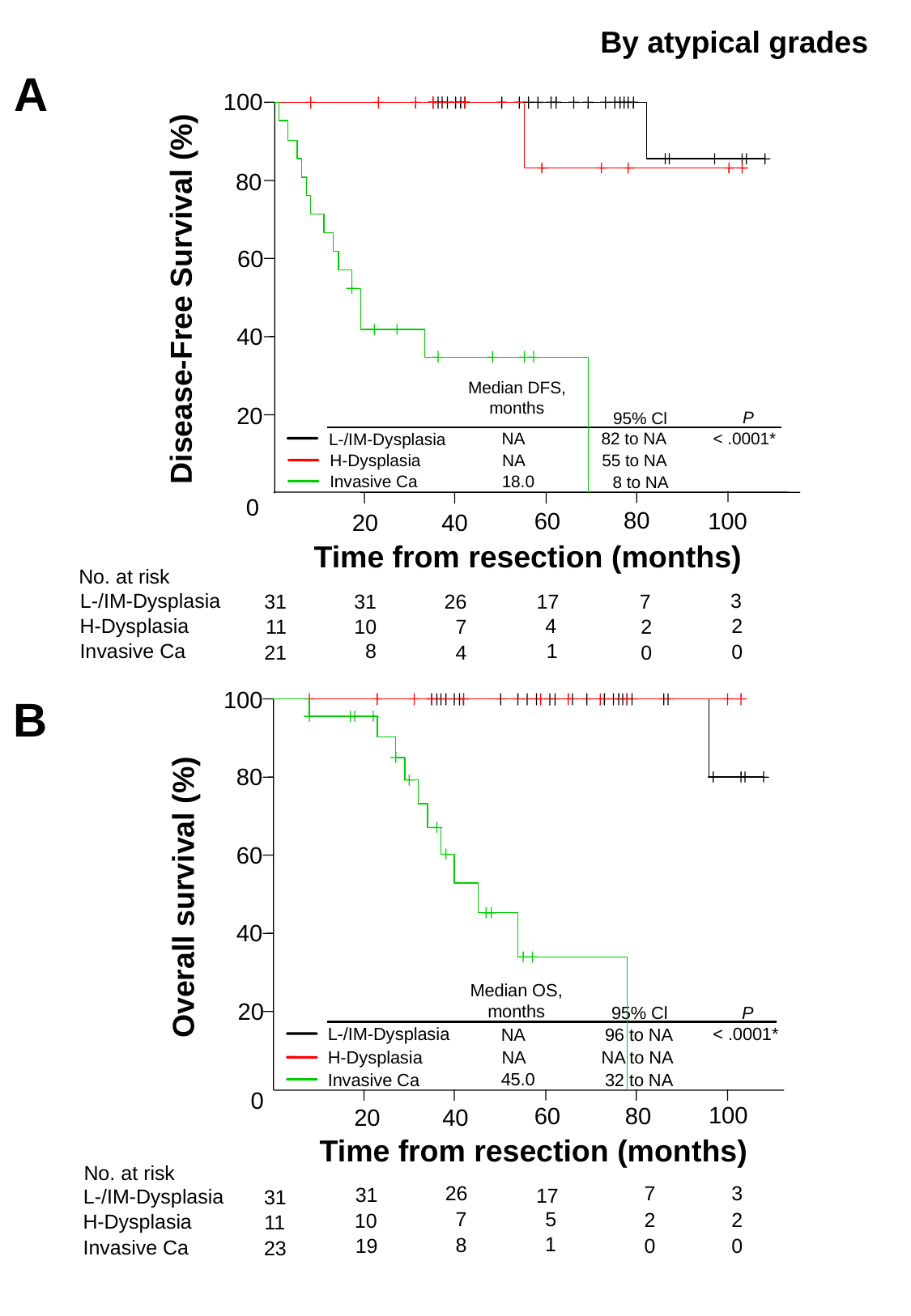

By atypical grades
A
100
80
60
Disease-Free Survival (%)
40
Median DFS,
months
20
P
95% Cl
NA
< .0001*
82 to NA
L-/IM-Dysplasia
NA
55 to NA
H-Dysplasia
18.0
Invasive Ca
8 to NA
0
80
60
100
20
40
Time from resection (months)
No. at risk
L-/IM-Dysplasia
3
31
31
26
17
7
4
H-Dysplasia
2
7
2
11
10
Invasive Ca
8
1
0
21
4
0
B
100
80
60
Overall survival (%)
40
Median OS,
months
P
95% Cl
20
< .0001*
L-/IM-Dysplasia
NA
96 to NA
NA
NA to NA
H-Dysplasia
45.0
32 to NA
Invasive Ca
0
100
80
60
20
40
Time from resection (months)
No. at risk
7
3
26
31
17
L-/IM-Dysplasia
31
5
7
2
2
10
H-Dysplasia
11
1
8
0
0
19
Invasive Ca
23

## Slide 2
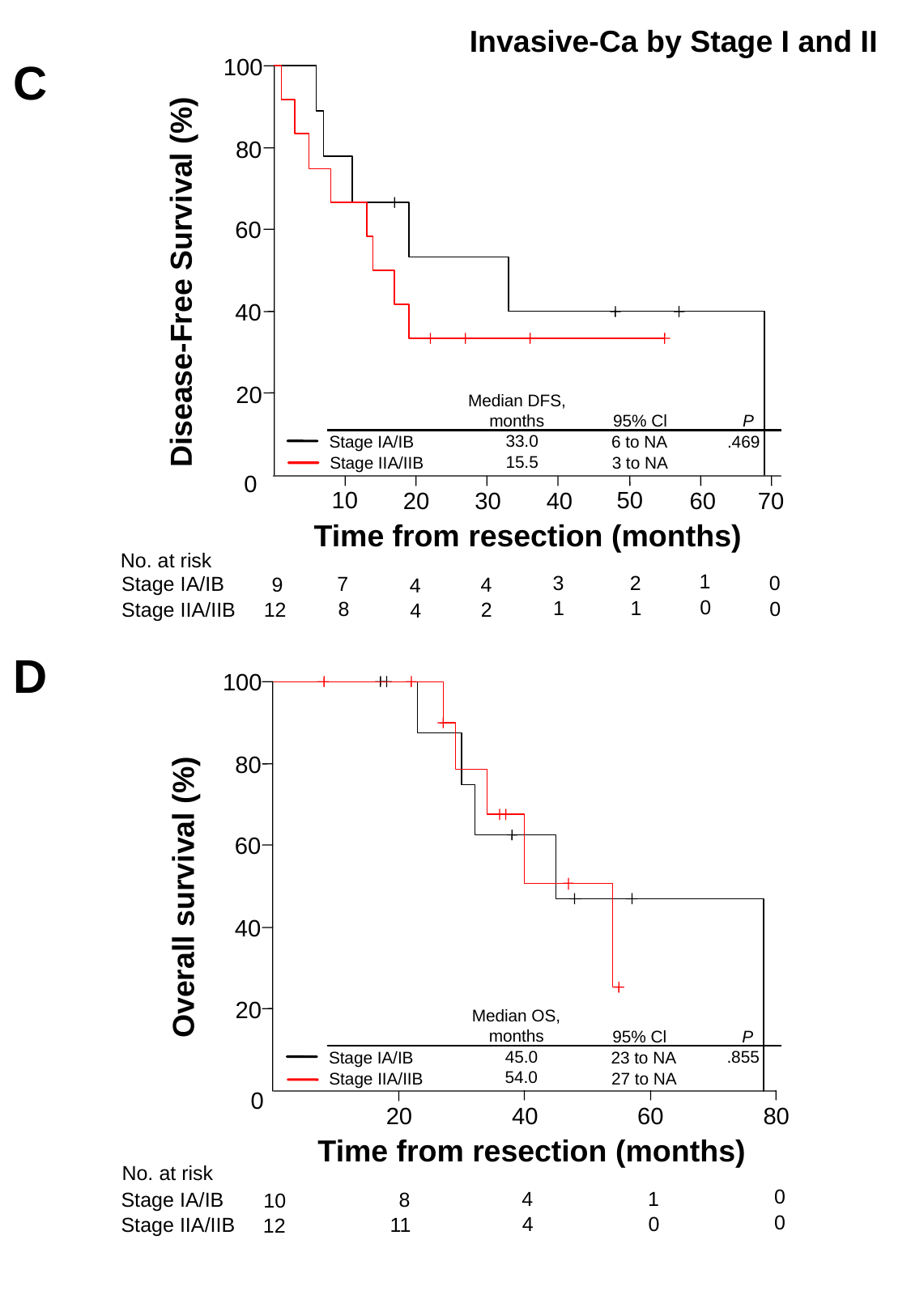

Invasive-Ca by Stage I and II
C
100
80
60
Disease-Free Survival (%)
40
20
Median DFS,
months
P
95% Cl
33.0
 .469
 6 to NA
Stage IA/IB
15.5
 3 to NA
Stage IIA/IIB
0
10
50
20
30
40
60
70
Time from resection (months)
No. at risk
1
3
2
0
7
Stage IA/IB
9
4
4
0
1
1
0
8
Stage IIA/IIB
2
12
4
D
100
80
60
Overall survival (%)
40
20
Median OS,
months
P
95% Cl
45.0
 .855
 23 to NA
Stage IA/IB
54.0
 27 to NA
Stage IIA/IIB
0
20
40
60
80
Time from resection (months)
No. at risk
0
4
1
8
Stage IA/IB
10
0
4
0
11
Stage IIA/IIB
12

## Slide 3
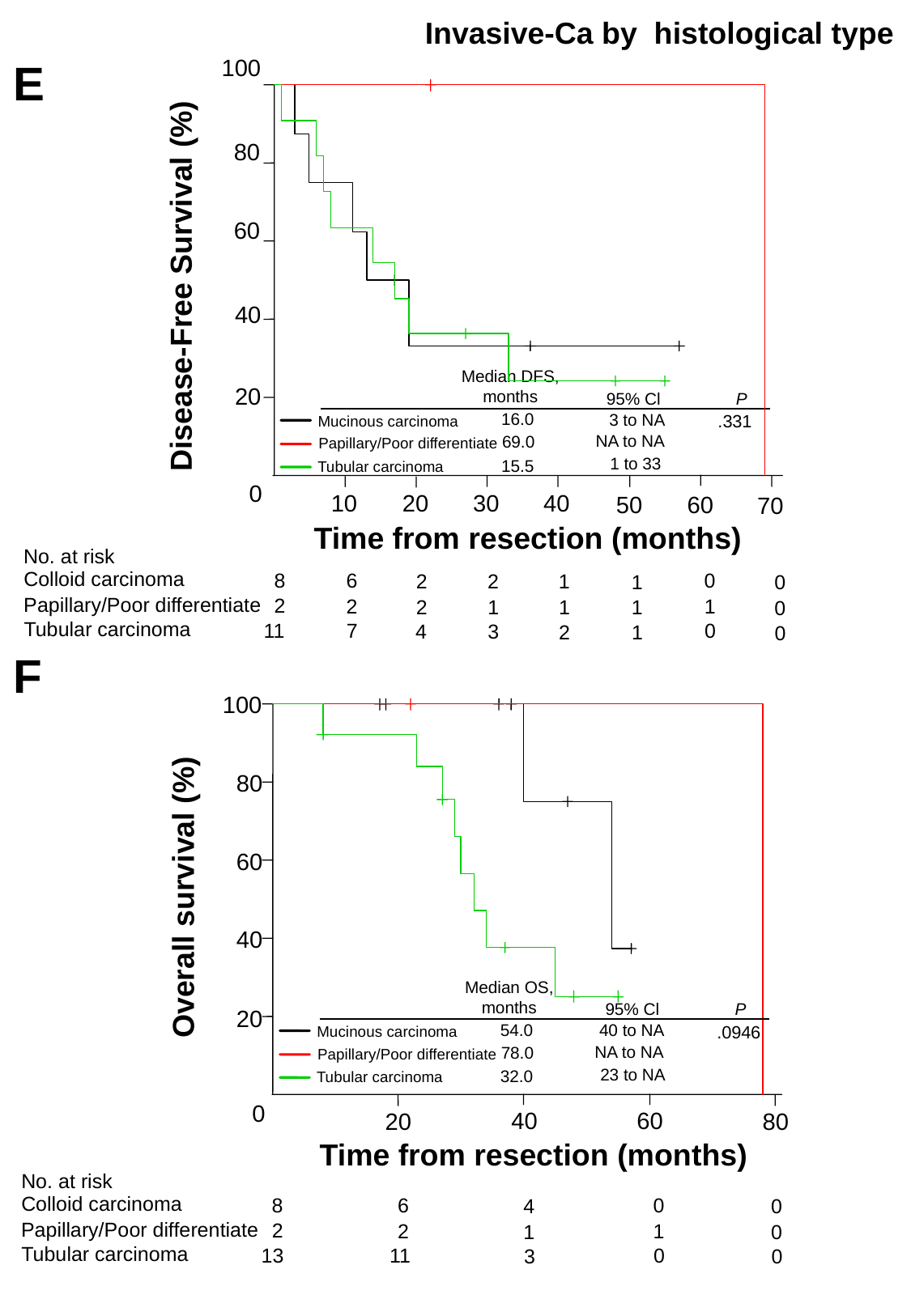

Invasive-Ca by histological type
E
100
80
60
Disease-Free Survival (%)
40
Median DFS,
months
P
95% Cl
16.0
3 to NA
.331
Mucinous carcinoma
Papillary/Poor differentiate
Tubular carcinoma
NA to NA
69.0
1 to 33
15.5
20
0
10
20
30
40
50
60
70
Time from resection (months)
No. at risk
Colloid carcinoma
8
0
6
2
2
1
1
0
Papillary/Poor differentiate
2
1
2
2
1
1
1
0
Tubular carcinoma
11
0
7
4
3
2
1
0
F
100
80
60
Overall survival (%)
40
Median OS,
months
P
95% Cl
54.0
40 to NA
.0946
Mucinous carcinoma
Papillary/Poor differentiate
Tubular carcinoma
NA to NA
78.0
23 to NA
32.0
20
0
40
60
20
80
Time from resection (months)
No. at risk
Colloid carcinoma
8
0
6
4
0
Papillary/Poor differentiate
2
1
2
1
0
Tubular carcinoma
13
0
11
3
0
