# Supplementary material for: Expansion of epigenetic alterations in EFEMP1 promoter predicts malignant formation in pancreatobiliary intraductal papillary mucinous neoplasms
Source: J Cancer Res Clin Oncol. 2016 Apr 19;142(7):1557–69. doi: 10.1007/s00432-016-2164-x (PMC4899496; doi:10.1007/s00432-016-2164-x)
Supplement: Supplementary file 2 — KRAS and GNAS mutations in IPMNs. (A) and (B) demonstrate the mutations occurring in KRAS codon 12 and GNAS codon 201, respectively. (C) Venn diagram of IPMNs with KRAS mutations, GNAS mutations, and concurrent KRAS and GNAS mutations. (D) Population of IPMNs with mutations in both genes, KRAS mutation alone, GNAS mutation alone, and wild type of both genes. Kaplan–Meier survival curves for disease-free survival (E and G), excluding a patient with remaining cancer at the resected margin, and overall survival (F and H) according to KRAS and GNAS mutation status, respectively. IPMN, intraductal papillary mucinous neoplasm; NA, not available (PPTX 126 kb) [file 432_2016_2164_MOESM2_ESM.pptx]

## Slide 1
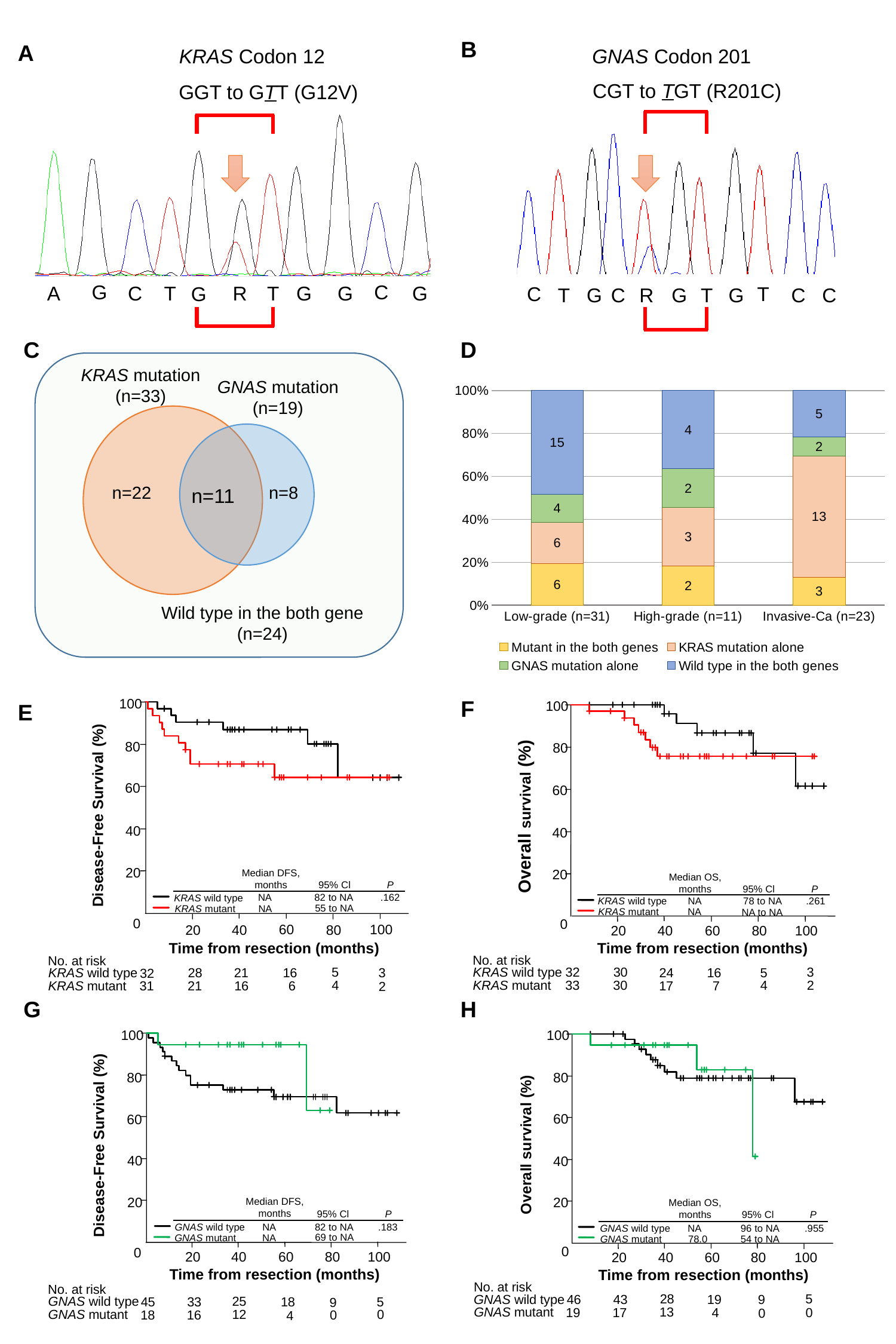

B
A
KRAS Codon 12
GNAS Codon 201
CGT to TGT (R201C)
GGT to GTT (G12V)
G
C
A
C
T
R
T
G
G
G
G
C
T
C
T
G
R
G
T
G
C
C
C
D
KRAS mutation (n=33)
GNAS mutation
(n=19)
### Chart
| Category | Mutant in the both genes | KRAS mutation alone | GNAS mutation alone | Wild type in the both genes |
|---|---|---|---|---|
| Low-grade (n=31) | 6.0 | 6.0 | 4.0 | 15.0 |
| High-grade (n=11) | 2.0 | 3.0 | 2.0 | 4.0 |
| Invasive-Ca (n=23) | 3.0 | 13.0 | 2.0 | 5.0 |
n=22
n=8
n=11
Wild type in the both gene
(n=24)
F
E
100
100
80
80
60
60
Overall survival (%)
Disease-Free Survival (%)
40
40
Median DFS,
months
95% Cl
P
NA
 .162
82 to NA
KRAS wild type
KRAS mutant
55 to NA
NA
20
20
Median OS,
months
95% Cl
P
KRAS wild type
NA
 .261
78 to NA
KRAS mutant
NA
 NA to NA
0
0
100
60
40
80
20
80
20
40
60
100
Time from resection (months)
Time from resection (months)
No. at risk
No. at risk
KRAS wild type
32
3
5
30
24
16
5
16
KRAS wild type
21
28
3
32
4
KRAS mutant
4
33
2
30
KRAS mutant
6
7
17
16
31
21
2
G
H
100
100
80
80
60
60
Overall survival (%)
Disease-Free Survival (%)
40
40
Median DFS,
months
Median OS,
months
20
20
95% Cl
P
95% Cl
P
.183
82 to NA
GNAS wild type
NA
 .955
96 to NA
NA
GNAS wild type
69 to NA
NA
GNAS mutant
GNAS mutant
54 to NA
78.0
0
0
20
40
100
80
60
80
20
40
60
100
Time from resection (months)
Time from resection (months)
No. at risk
No. at risk
5
28
46
43
GNAS wild type
19
9
GNAS wild type
25
45
5
33
18
9
GNAS mutant
13
0
19
17
4
0
GNAS mutant
12
0
18
16
0
4
